# Prevalence of frailty among patients with inflammatory bowel disease and its association with clinical outcomes: a systematic review and meta-analysis

**DOI:** 10.1186/s12876-022-02620-3

**Published:** 2022-12-22

**Authors:** Xiangting Huang, Mengmeng Xiao, Benyue Jiang, Xiuzheng Wang, Xiaoyu Tang, Xiao Xu, Ying Chen, Shixuan Wang, Songbo Yan, Siyi Wang, Jiang Wang, Pinglan Zhang

**Affiliations:** 1 Department of Medicine, Jinggangshan University, Ji’an, Jiangxi Province China; 2 Department of Philosophy, University of Jena, Jena, Germany

**Keywords:** Frailty, Inflammatory bowel disease, Meta-analysis

## Abstract

**Background:**

Studies have reported frailty as an independent risk factor of mortality in patients with inflammatory bowel disease (IBD). However, no systematic review and meta-analysis has been conducted to determine the relationship of frailty and IBD. We aimed to investigate the prevalence of frailty in patients with IBD and the impact of frailty on the clinical prognosis of these patients.

**Methods:**

We systematically searched PubMed, Ovid (Medline), Embase, Web of Science, and Cochrane Library from database inception until October 2022. This systematic review included observational studies describing IBD and frailty. We performed meta-analysis for the frailty prevalence in patients with IBD. We analyzed primary outcomes (mortality) and secondary outcomes (infections, hospitalizations, readmission, and IBD-related surgery).

**Results:**

Nine studies with a total of 1,495,695 participants were included in our meta-analysis. The prevalence of frailty was 18% in patients with IBD. The combined effect analysis showed that frail patients with IBD had a higher risk of mortality (adjusted hazard ratio = 2.25, 95% confidence interval: 1.11–4.55) than non-frail patients with IBD. The hazard ratio for infections (HR = 1.23, 0.94–1.60), hospitalizations (HR = 1.72, 0.88–3.36), readmission (HR = 1.21, 1.17–1.25) and IBD-related surgery (HR = 0.78, 0.66–0.91) in frail patients with IBD.

**Conclusions:**

We demonstrated that frailty is a significant independent predictor of mortality in patients with IBD. Our work supports the importance of implementing frailty screening upon admission in patients with IBD. More prospective studies are needed to investigate the influence of frailty on patients with IBD and improve the poor prognosis of patients with frailty and IBD.

**Supplementary Information:**

The online version contains supplementary material available at 10.1186/s12876-022-02620-3.

## Introduction

Frailty refers to a clinically identifiable state of increasing vulnerability in older adults. Sarcopenia is defined by low muscle strength and low muscle quantity or quality. Age-related declines in function and physiological reserves of multiple organ systems can lead to frailty [1]. Frailty may occur in patients of any age but is more common in elderly patients [2]. Frailty is common in community and clinical settings and can predict many various adverse health outcomes, including mortality [3], disability [4], worsening mobility [5], loneliness [6], falls [7], fractures [8], hospitalization [8], lower quality of life [9], depression [10], dementia [11], cognitive decline [12], and nursing home admission [13]. The connection between mortality and frailty has been verified in many studies and involves many settings and sub-populations [14–16].

Inflammatory bowel disease (IBD) is a chronic systemic inflammatory condition and includes ulcerative colitis (UC), Crohn disease (CD), and indeterminate colitis. IBD can cause various symptoms, such as diarrhea, abdominal discomfort, nausea, vomiting, and joint pain [17]. Between 1990 and 2017, the number of individuals with IBD rose from 3.7 million to over 6.8 million, and the global prevalence of IBD increased by 85.1% [18]. The burden of IBD in elderly individuals is increasing with population aging [19]. Approximately 25%–35% of individuals with IBD are over age 60 years [20, 21]. In this age group, the incidence rates of UC are almost universally higher than the rates of CD. The incidence rates of UC range from 1.8 to 20 per 100,000 in the United States and Europe; the incidence rates in the Asia–Pacific region are much lower. The incidence rates of CD range from 1–10 per 100,000 in Europe and more than 50 per 100,000 in New Zealand; the incidence of CD is much lower in the rest of the Asia–Pacific region [22].

Population-based studies indicate that mortality is higher among patients with IBD than among the general population [23, 24]. Thus, it is important to identify the mediators of IBD risk. One study demonstrated that frailty was more prevalent in approximately one-third of hospitalized adults with IBD [25]. Frailty is a substantial physiologic driver of IBD outcomes, including therapy-related infection complications, death, and hospital readmission, according to certain studies [26–28]. However, no in-depth analysis has been conducted regarding whether frailty in patients with IBD and all-cause mortality are related. We therefore carried out this systematic review and meta-analysis to investigate how frailty affects outcomes of patients with IBD.

## Method

We followed the Meta-analysis Of Observational Studies in Epidemiology (MOOSE) guideline in this systemic review [29].

### Search strategy

We comprehensively searched several databases and websites, including PubMed, Cochrane, Medline (via Ovid), Embase (via Ovid), and Web of Science, from inception of each database to July 28, 2021. We conducted an updated search in October 2022. Our search strategy included suitable Medical Subject Headings (Mesh) and free-texts words to identify frailty and IBD; the detailed search strategy and search terms can be found in the supplement. We also manually searched the reference lists of all identified studies.

### Study selection

Two researchers independently screened all titles and abstracts to confirm the eligibility of relevant studies. Next, the full texts of studies warranting further investigation were assessed independently by two researchers. We resolved all disagreements in discussion with a third researcher until consensus was reached. We used the following inclusion criteria for studies: (1) the study population was mainly patients with IBD; (2) frailty was a risk factor; (3) the prevalence of frailty in the study population was recorded; (4) data on clinical outcomes of frailty in patients with IBD were available in the included studies; (5) the original research was observational research. We excluded the following studies: (1) studies that did not have a cohort of patients with IBD grouped according to frailty and non-frailty; (2) no data on patients with IBD were reported separately in the study; (3) randomized controlled trials (RCTs), reviews, and conference abstracts.

### Data extraction

Two researchers independently extracted and reviewed the data. We collected the following information for each study: author, country, study design, percentage of men, mean age, sample size, frailty criteria, assessment of frailty, type of IBD, follow-up period (cohort studies only), primary outcome (mortality), secondary outcomes (infections, hospitalizations, readmission, and IBD-related surgery), and prevalence of frailty.

### Assessment of quality

We used the Newcastle–Ottawa Scale (NOS) to evaluate the quality of the included studies. The NOS is a nine-point scale with three domains. Four points were allocated for study population selection, two points for comparability, and three points for outcome. We considered studies with NOS ≥ 8 to be of high quality. The comparability score depends on the relevant confounding factors that are corrected. One point was obtained if only correcting confounding factors affecting the outcome or only correcting other critical confounding factors. If both are corrected, two points were given; no score was given if neither is corrected. Two independent investigators discussed any disagreement in the quality assessment.

### Statistical analysis

We performed a meta-analysis for the prevalence of frailty in patients with IBD using Stata 12 software (StataCorp LLC, College Station, TX, USA). According to the heterogeneity of relevant studies, we selected corresponding effects models. We used the chi-squared test and the I^2^ statistic to identify heterogeneity [30]. When the former test had a value of p ≤ 0.05 and the latter a value of I^2^ ≥ 50%, this indicated significant heterogeneity. We used a forest plot to estimate the effect size and 95% confidence intervals (CIs). Because the content in data extraction can lead to heterogeneity, we used meta-regression to calculate more reliable combined statistics and analyzed the sources of heterogeneity after subgroup analysis. We conducted sensitivity analyses after removing studies that did not report effect size in the meta-analysis of clinical outcome for patients with IBD. We used Egger’s test and Begg’s test to assess publication bias (p < 0.05) by visually inspecting the funnel plots [31, 32]. Additionally, we used the trim-and-fill method to explain publication bias.

## Results

### Study selection

**Fig. 1:**
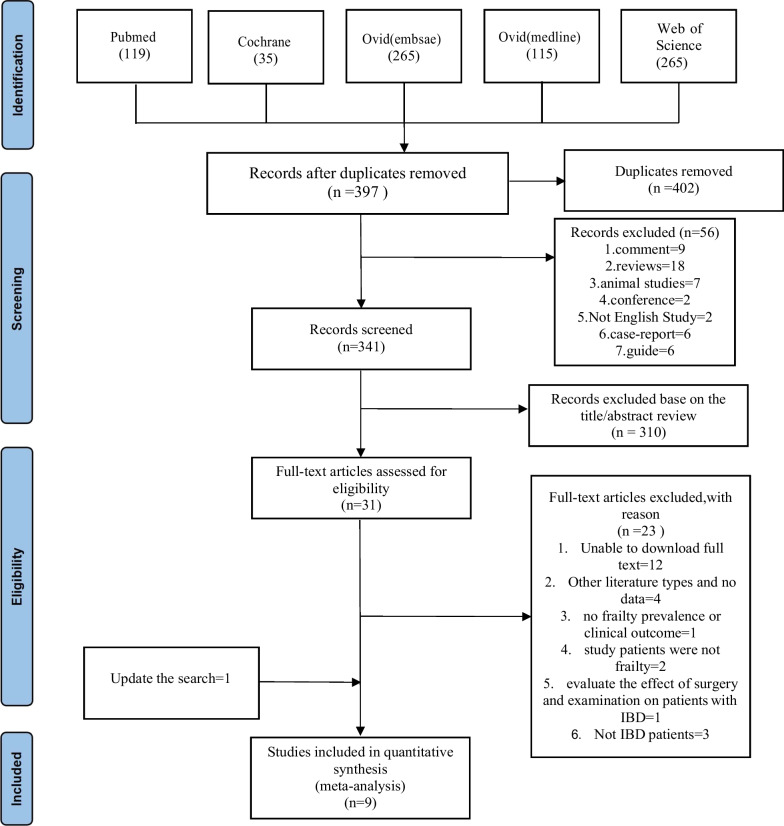
The flow diagram of studies selection

In our initial search (July 2021), we identified 799 articles in PubMed, Cochrane, Embase, Medline, and Web of Science (Fig. 1). Of these, we excluded 402 duplicate articles and 56 ineligible publications (nine comments, 18 reviews, seven animal studies, two conferences, two non-English studies, six case reports, six guidelines, and six RCTs). We checked the title and abstract of each remaining article in preliminary screening according to the established criteria, and identified 31 publications related to the research topic. Among them, 12 were excluded owing to unavailability of the complete text, 11 of the 19 articles with full text were excluded after reading the entire text in detail, according to the predetermined criteria. Additionally, we performed an updated database search from July 2021 to October 2022. Finally, this study included nine publications.

### Summary of studies

The included studies comprised seven retrospective cohort studies and two prospective cohort studies (Table 1). The study populations in the original research were from well-documented databases, including two from a cohort of 11,001 patients with IBD, one from an administrative claims database, two from the Nationwide Readmissions Database, two from the American College of Surgeons National Surgical Quality Improvement Program database, one from a cohort of patients with CD or UC using electronic health records, and one from the Nationwide Patient Register database. Of the nine enrolled studies, seven both CD and UC in IBD types, one included only CD and one included only UC. The nine studies included a total of 1,495,695 participants, and 175,154 were identified as having frailty at the time of inclusion, with a frailty rate of 11.71%. Table 1 shows the overall traits of the included studies.

**Table 1:** Characteristics of the included studies

Author year	Country	Study design	Male (%)	Age (mean/SD)	Sample size	Type of IBD	Follow-up	Participants	Definition of frailty/frailty Criteria	Frailty assessment (cut-off)	Prevalence of frailty	Outcome	Quality score
Alexander 2020 [25]	USA	Retrospective cohort study	42.89	53.35 ± 19.34	47,402	CD and UC	10 months	The Nationwide Readmissions Database (NRD) 2013	The Hospital Frailty Risk Score ICD-9-CM	Medium frailty risk (score 5–15) and high frailty risk (score > 15)	15,507 (32.71%)	Mortality, readmission, hospitalization, and IBD-related surgery	9
Bharati Kochar 2021 [26]	USA	Retrospective cohort study	46.91	34.37 ± 17.04	1210	CD and UC	1 year	An electronic health record (EHR)-based cohort of patients with CD or UC	A validated claims-based frailty index (CFI)	Normalized CFI frailty score ≥ 3	189 (15.6%)	NA	7
Bharati Kochar 2020 [27]	USA	Prospective cohort study	47	46.82 ± 21.58	11,036	CD and UC	8.61 ± 8.48 years	Used a previously well-described cohort of 11,001 patients with IBD	International Classification of Disease ICD-10 codes	The presence ≥ 1 ICD code	675 (6%)	NA	9
Bharati Kochar 2020 [28]	Boston	Retrospective cohort study			3975	CD and UC		A cohort study of 11,001 patients with IBD from Partners Healthcare system	Frailty Risk Score, a International Classification of Disease codes (ICD-9,10 codes)	Presence of at least one frailty related ICD-9 code in the 2 y prior to the first electronic prescription		NA	9
			46	36.95 ± 18.58	1299		8.59 ± 7.47 years	Anti-TNF Study Population			68 (5.23%)		
			48	39.58 ± 20.24	2676		8.94 ± 7.59 years	Immunomodulator (IMM) Study population			212 (7.92%)		
Adam S.Faye 2021 [33]	USA	Retrospective cohort study	43.21	all age	1,405,529	CD and UC	30 days	Weighted Nationwide Readmission Database(NRD)	A validated ICD-9-CM codes	Patients with any of the aforementioned ICD-9-CM codes were considered frail	152,974 (10.9%)	NA	8
Siddharth Singh 2020 [34]	USA	Retrospective cohort study	50.50	41.57 ± 15.37	5987	CD and UC	14.45 ± 13.32 years	An administrative claims database	The Hospital Frailty Risk Score (ICD-9-CM; ICD-10-CM)	Frailty risk score 5 or higher	2350 (39.3%)	Serious and/or opportunistic infections†	8
Joshua 2021 [35]	Baltimore	Retrospective cohort study	46.76	> 18	9023	CD	30 days	The American College of Surgeons National Surgical Quality Improvement Program (ACS NSQIP)	Simplified Frailty Index (SFI) score	A score of 0 is the least frail, whereas 5 is the most frail	1610 (17.8%)‡	NA	8
Edwin telemi 2018 [53]	Arizona	Retrospective cohort study	54.30	46 ± 19.30	943	UC	30 days	The National Surgical Quality and Improvement Program (NSQIP) cross-institutional database	Modified frailty index (MFI)	MFI > 0	305 (32.34%)	NA	7
Bharati Kochar 2022 [54]	Sweden	Prospective cohort study	48	71 ± 8	10,590	CD and UC	5 ± 3 years	Nationwide Patient Register (NPR) Database	The Hospital Frailty Risk Score (HFRS)	Low frailty risk(score 0–5) and high frailty risk (score > 5)	1264(12%)	Mortality and hospitalization	8

Abbreviation: IBD, inflammatory bowel disease; CD, Crohn’s disease; UC, ulcerative colitis

†Serious and/or opportunistic infections (defined as infection requiring hospitalization), 520 (8.69%) patients developed serious infection

‡1610 (17.8%) was SFI > 0; 7413 (82.2%) was SFI = 0

### Study quality

The included publications were all cohort studies without randomized controlled studies. Quality evaluation was conducted using the NOS scale. The overall quality of the studies was good, with scores ranging between 7 and 9. Studies with NOS scores ≥ 8 were deemed to be high quality. After being included in the research participation score, seven articles were considered high quality, and two articles were rated with 7 points. The quality score was also good. Additional file 1: Table S1 shows the quality assessment.

### Prevalence of frailty in patients with IBD

**Fig. 2:**
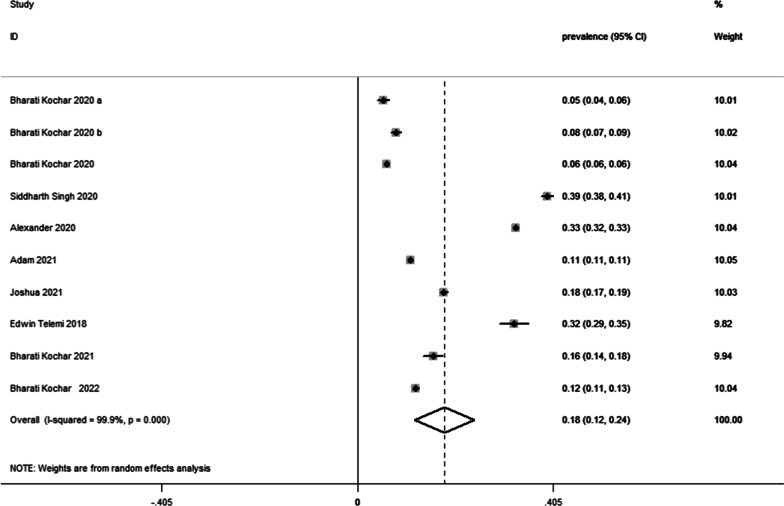
Prevalence of frailty in IBD patients

Among 1,495,695 study participants in the nine included studies, a total of 175,154 patients had frailty. We performed a meta-analysis of the frailty prevalence in the populations included in the nine studies, and the final pooled prevalence was 18% (95% CI: 12–24%, p = 0.000) (Fig. 2). Because I^2^ = 99.9% and p < 0.001, we choose the random-effects model to assess prevalence.

We performed a subgroup analysis of study design, male proportion, sample size, participants, frailty criteria, and follow-up (Additional file 1: Table S2). Meta-regression showed that the sex ratio was a possible source of heterogeneity (β = − 0.223, standard error = 0.068, p = 0.011) (Additional file 1: Table S3). In the subgroup by sex, the prevalence of male proportion ≥ 50% was 0.36 (95% CI: 0.29–0.43) and that of male proportion < 50% was 0.14 (95% CI: 0.07–0.20). Additionally, we performed subgroup analysis for age, but there were insufficient data to confirm that age was responsible for differences in the prevalence of frailty among patients with IBD (Additional file 1: Table S2).

Our funnel plot was symmetrical (Additional file 1: Fig. S1), and the Begg’s test (p = 0.371) and Egger’s test (p = 0.242) also indicated no publication bias. Additionally, sensitivity analysis showed no significant change in the pooled results after excluding individual studies one by one. This proved that the results were stable (Additional file 1: Fig. S2).

### Primary outcome

In the nine included studies, we focused mainly on clinical outcomes associated with frailty factors in patients with IBD. The primary outcome is mortality in our study. We extracted the hazard ratio (HR) of mortality from the included studies and subsequently performed corresponding meta-analysis. Two studies [25, 36] reported mortality in frail IBD patients (n = 16,771) and in non-frail IBD patients (n = 41,221). Combined effect analysis showed that frailty increased the risk of mortality in patients with IBD compared with patients who had IBD without frailty (adjusted HR 2.25, 95% CI: 1.11–4. 55).

### Secondary outcomes

The secondary outcomes included infection, hospitalization, readmission, and IBD-related surgery. In this study, we did not have sufficient data to confirm that frailty increases the risk of infection (adjusted HR 1.23, 95% CI 0.94–1.60) and hospitalization (adjusted HR 1.72, 95% CI: 0.88–3.36) in patients with IBD. One of the studies [25] reported that frail patients with IBD were more susceptible to readmission treatment (adjusted HR 1.21, 95% CI: 1.17–1.25), and frailty reduced the risk of IBD-related surgery (adjusted HR 0.78, 95% CI: 0.66–0.91) (Table 2).

**Table 2:** Impact of frailty on clinical outcomes in patients with IBD

Outcome	Study	Sample size	Effect size	P(z-text)	I^2^ (%)
Mortality	Alexander [25], Kochar [54]	57,992	2.25 (1.11, 4.55)	< 0.05	98.1
Infections	Singh [34]	5987	1.23 (0.94, 1.60)	0.124	76.0
Hospitalizations	Alexander [25], Kochar [54]	57,992	1.72 (0.88, 3.36)	0.114	99.5
Readmission	Alexander [25]	47,402	1.21 (1.17, 1.25)	< 0.05	–
Risk of IBD-related surgery	Alexander [25]	47,402	0.78 (0.66, 0.91)	< 0.05	–

## Discussion

In our study, we found that frailty was common in patients with IBD. Compared with the general population, patients with IBD have a higher prevalence of frailty [36]. Our results also showed that frailty was associated with increased mortality risk in patients with IBD. However, frailty reduces the risk of IBD-related surgery perhaps because doctors do not recommend surgical treatment for frail patients with IBD. We included high-quality studies, adjusted the corresponding confounding factors, and conducted subgroup analysis and meta-regression analysis to deal with the heterogeneity among different studies such that our study findings were stable and reliable.

In the long-term process of the inflammatory bowel disease disease, there is a decline in the reserves of multiple physiological systems and a chronic increase in the level of circulating inflammatory markers. In a cross-sectional study of 117 participants aged 62–95 years, frailty was associated with increased serum interleukin 6, soluble tumor necrosis factor (TNF)-αreceptor-I as well as soluble TNF-αreceptor-II, suggesting an association of frailty with circulating markers of inflammation [37]. Another study showed that signs of frailty and circulatory inflammation are associated with the incidence of multiple complications and infections [38]. Immunosuppressive drugs are often used to treat IBD. Patients using such drugs have a weak immune response, which is one reason for frailty and an increased risk of infections [39]. Additionally, frailty patients have a reduced ability to recover, and an increased probability of complications [40], which may make the treatment of patients with IBD more difficult [41] and reduce the possibility of recovery to baseline, increasing the chance of hospitalization [42–44]. Frailty is a stress response owing to the imbalance of immune, endocrine, and energy response systems, Frailty is dynamic, but its specific biological basis has not been fully clarified. According to the existing evidence, frailty is related to increased systemic inflammatory markers [45–47]. Thus, there is reason to believe that the prevalence of frailty in patients with IBD is higher than in those without IBD.

Frailty is an important measure of functional status [48]. It is considered a biological syndrome with reduced reserves caused by the cumulative decline of multiple physiological functions or a risk index based on the accumulation of health defects [2, 49]. Its relevant definitions include diagnoses related to dementia, visual impairment, bedsores, fecal incontinence, social support needs, action capability, and nutritional problems [50]. Although many well-validated methods for assessing frailty have been developed in the general adult and elderly populations, there are no fully validated tools for assessing frailty in the population of patients with IBD [51, 52]. The nine cohort studies included in this review used several different frailty assessment tools. Surprisingly, even with the same frailty assessment tool, the cut-off in frailty assessment differed among cohorts. This may be because there is no gold standard to assess frailty to date. Because of differences in the data sources of the original research, different studies used different assessment tools and diagnostic criteria for frailty, which may be one reason for the large heterogeneity in our included studies. This also suggests that more research is needed to improve diagnostic criteria as well as more systematic evaluation of frailty in patients with IBD.

Bedard et al. [54] published a systematic review that described the clinical outcomes of frailty in patients with IBD. Our study is the first systematic assessment and meta-analysis of frailty in patients with IBD in recent years. We described the relationship between frailty and various clinical outcomes of IBD diseases, including mortality, infection, readmission, hospitalization, and IBD-related surgery. This work further supports frailty as an important prognostic factor, independent of age and comorbidity. Frailty is a crucial risk stratification factor in patients with IBD to help identify particularly high-risk adverse events in this population and determine the best treatment for improving outcomes in frail patients with IBD. Our work also supports the value of clinical frailty screening in patients with IBD at admission to better treat the disease.

Our study adds to the existing literature on frail patients with IBD and has several advantages. An important strength is that we examined the prevalence of frailty in patients with IBD and the relationship between frailty and clinical outcomes in these patients; this is more efficient and valuable than research of single effect estimates. We found a higher prevalence of frailty and greater risk of mortality in patients with IBD. This highlights the need for clinicians to pay greater attention to frailty in patients with IBD and to intervene early in these patients to prevent adverse outcomes, reduce health care expenditures, and increase the life expectancy of patients with IBD. Another advantage of this study is the inclusion of a large population of patients with IBD. We performed a pooled analysis of prevalence using a large sample size. This facilitates more accurate estimates of the prevalence of frailty in patients with IBD. Our study is limited in that we did not perform subgroup analysis of clinical outcomes in the included cohort studies because of insufficient data on the impact of frailty on clinical outcomes in patients with IBD. Also, the small number of included publications may affect the credibility of the results. Further studies are warranted to confirm our results in the future.

## Conclusions

In the present meta-analysis of nine cohort studies based on the population with IBD, we found that frailty is increasingly prevalent among patients with IBD and that frailty is a significant and independent predictor of mortality in these patients. Our work highlights the value of implementing frailty screening in patients with IBD on admission. Further research to identify gold standard criteria for diagnosing and evaluating frailty is needed, as well as prospective studies focused on the impact of frailty in patients with IBD, to improve the poor prognosis of frail patients with IBD.

## Supplementary Information

**Additional file 1: Table S1:** The quality of included studies by NOS scale (cohort study). **Table S2:** Subgroup analysis for prevalence of frailty in patients with IBD. **Table S3:** Meta-regression for the prevalence of frailty. **Figure S1:** Funnel plot of prevalence of frailty in patients with IBD. **Figure S2:** Sensitivity analysis of the prevalence of frailty in patients with IBD.

## Data Availability

All data generated or analyzed during this study are included in this published article and its supplementary information files.
