# Supplementary material for: Prevalence of frailty among patients with inflammatory bowel disease and its association with clinical outcomes: a systematic review and meta-analysis
Source: BMC Gastroenterol. 2022 Dec 22;22:534. doi: 10.1186/s12876-022-02620-3 (PMC9773593; doi:10.1186/s12876-022-02620-3)
Supplement: Supplementary file 1 — Additional file 1: Table S1: The quality of included studies by NOS scale (cohort study). Table S2: Subgroup analysis for prevalence of frailty in patients with IBD. Table S3: Meta-regression for the prevalence of frailty. Figure S1: Funnel plot of prevalence of frailty in patients with IBD. Figure S2: Sensitivity analysis of the prevalence of frailty in patients with IBD. [file 12876_2022_2620_MOESM1_ESM.docx]

***Supplementary information***

| Table S1 The quality of included studies by NOS scale (cohort study). | | | | | | | | | | | | |
| --- | --- | --- | --- | --- | --- | --- | --- | --- | --- | --- | --- | --- |
|  | **Selection (1)** | | | | | **Comparability (2)** | | **Outcome (3)** | | | **S**core | |
| **Study** | Representativeness of the intervention cohort | Selection of the non intervention cohort | Ascertainment of intervention | Demonstration that outcome of interest was not present at start of study | Whether significant confounders were adjusted for | | Assessment of outcome | | Was follow up long enough for outcomes to occur | Adequacy of follow up of cohorts | | Total points |
| Bharati Kochar 2020 [28] | ✮ | ✮ | ✮ | ✮ | ✮✮ | | ✮ | | ✮ | ✮ | | 9 |
| Edwin Telemi 2018 [53] | ✮ | ✮ | ✮ | ✮ | ✮ | | ✮ | |  | ✮ | | 7 |
| Bharati Kochar 2020 [27] | ✮ | ✮ | ✮ | ✮ | ✮✮ | | ✮ | | ✮ | ✮ | | 9 |
| Siddharth Singh 2020 [34] | ✮ | ✮ | ✮ | ✮ | ✮ | | ✮ | | ✮ | ✮ | | 8 |
| Alexander 2020 [25] | ✮ | ✮ | ✮ | ✮ | ✮✮ | | ✮ | | ✮ | ✮ | | 9 |
| Bharati Kochar 2021 [26] | ✮ | ✮ | ✮ | ✮ |  | | ✮ | | ✮ | ✮ | | 7 |
| Adam S.Faye 2021 [33] | ✮ | ✮ | ✮ | ✮ | ✮✮ | | ✮ | |  | ✮ | | 8 |
| Joshua 2021[35] | ✮ | ✮ | ✮ | ✮ | ✮✮ | | ✮ | |  | ✮ | | 8 |
| Bharati Kochar 2022[36] | ✮ | ✮ | ✮ | ✮ | ✮✮ | | ✮ | |  | ✮ | | 8 |

| Table S2 Subgroup analysis for prevalence of frailty in patients with IBD. | | |
| --- | --- | --- |
| **Variable** | **Random-effect model** | **Heterogeneity** |
| Study design |  |  |
| Prospective cohort study | 0.09 (0.03, 0.15) | I^2^=99.6%, P<0.001 |
| Retrospective cohort study | 0.20 (0.11, 0.29) | I^2^=99.9%, P<0.001 |
| Male% |  |  |
| ≥50% | 0.36 (0.29, 0.43) | I^2^=94.4%, P<0.001 |
| <50% | 0.14 (0.07, 0.20) | I^2^=99.9%, P<0.001 |
| Age† |  |  |
| ≥65 | 0.26 (-0.02, 0.53) | I^2^=100%, P<0.001 |
| <65 | 0.12 (0.04, 0.20) | I^2^=100%, P<0.001 |
| Sample size |  |  |
| >10000 | 0.15 (0.05, 0.26) | I^2^=100.0%, P<0.001 |
| <10000 | 0.20 (0.09, 0.30) | I^2^=99.8%, P<0.001 |
| Participants |  |  |
| Cohort of 11001 patients with IBD | 0.06 (0.05, 0.08) | I^2^=85.8%, P=0.001 |
| NRD Database | 0.22 (0.004, 0.43) | I^2^=100.0%, P<0.001 |
| ACS NSQIP database | 0.25 (0.11, 0.39) | I^2^=98.8%, P<0.001 |
| Administrative claims database | 0.39 (0.38, 0.41) | - |
| Electronic health record (EHR)-based cohort | 0.16 (0.14, 0.18) | - |
| NPR Database | 0.12 (0.11, 0.13) | - |
| Definition of frailty |  |  |
| Hospital Frailty Risk Score | 0.16 (0.09, 0.24) | I^2^=100.0%, P<0.001 |
| Simplified Frailty Index (sFI) score | 0.18 (0.17, 0.19) | - |
| Modified frailty index (mFI) | 0.32 (0.29, 0.35) | - |
| Claims-based frailty index (CFI) | 0.16 (0.14, 0.18) | - |
| Follow-up |  |  |
| <1year | 0.23 (0.10, 0.37) | I^2^=99.8%, P<0.001 |
| ≥1year | 0.14 (0.06, 0.22) | I^2^=100.0%, P<0.001 |

†: The data were obtained from three studies Alexander 2020, Adam 2021, and Joshua 2021.

Abbreviations: IBD, inflammatory bowel disease; NRD, Nationwide Readmission Database; ACS NSQIP, The American College of Surgeons National Surgical Quality Improvement Program; NPR, Nationwide Patient Register.

| Table S3 Meta-regression for the prevalence of frailty. | | | |
| --- | --- | --- | --- |
| **Variable** | **β (95% CI)** | **SE** | **p** |
| Study design: retrospective cohort study vs prospective cohort study | 0.112 (-0.109, 0.333) | 0.096 | 0.275 |
| Male%: <50% vs ≥50% | -0.223 (-0.380, -0.067) | 0.068 | 0.011 |
| Age(mean): <65years vs other | 0.005 (-0.186, 0.197) | 0.083 | 0.950 |
| Sample size: <10000 vs >10000 | 0.043 (-0.149, 0.235) | 0.083 | 0.622 |
| Participants: ACS NSQIP database vs other | 0.088 (-0.140, 0.317) | 0.099 | 0.400 |
| Definition of frailty: Hospital Frailty Risk Score vs other | -0.056 (-0.260, 0.148) | 0.088 | 0.545 |
| Follow-up: ≥1year vs <1year | 0.091 (-0.090, 0.271) | 0.078 | 0.281 |

Abbreviations: ACS NSQIP, The American College of Surgeons National Surgical Quality Improvement Program.

Figure S1 Funnel plot of prevalence of frailty in patients with IBD.

***
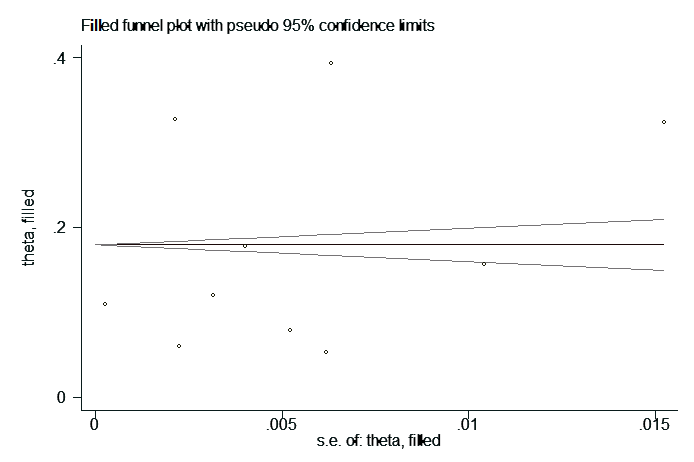
***

Figure S2 Sensitivity analysis of the prevalence of frailty in patients with IBD.

**
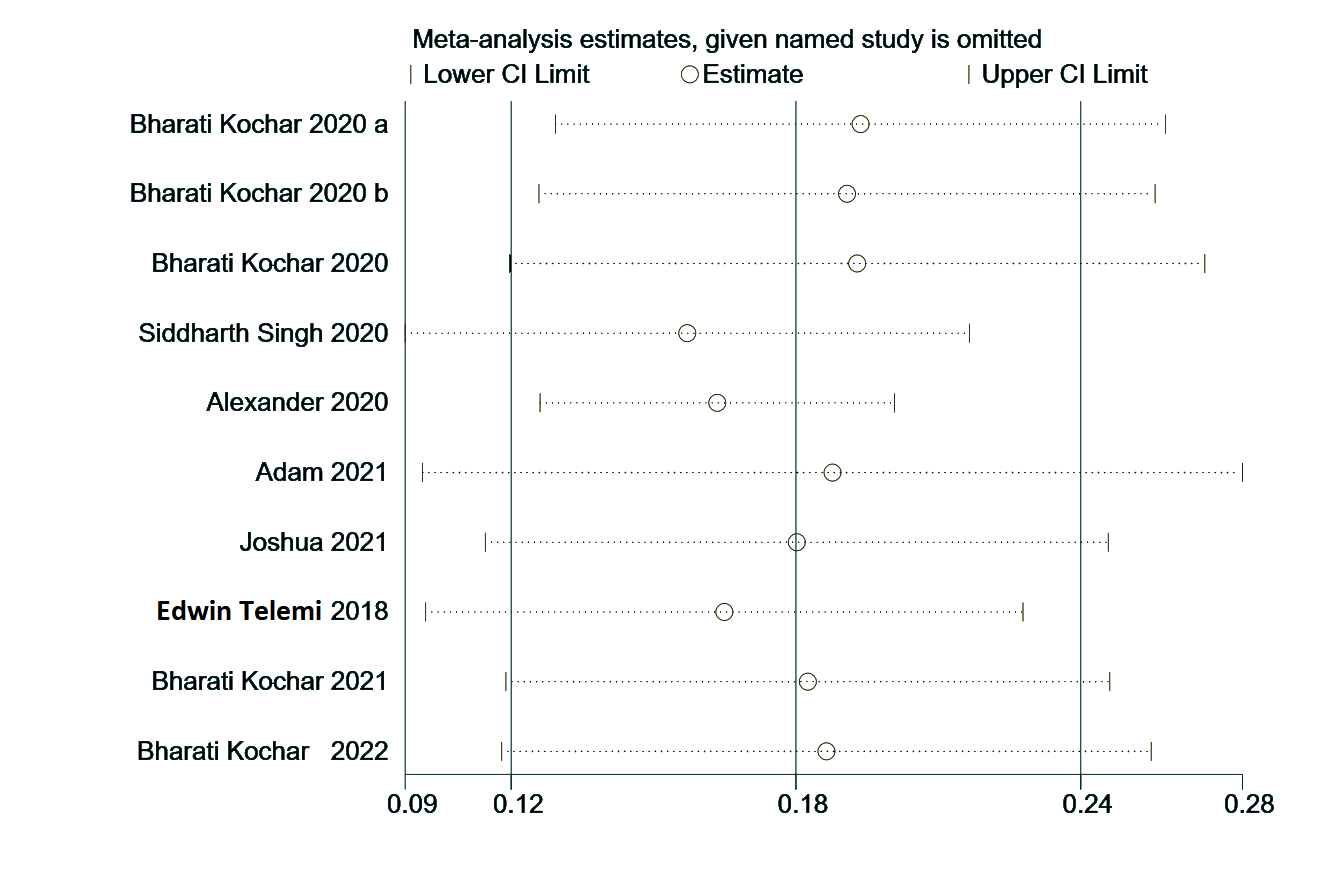
**
